# Undergraduate courses of evidence-based medicine in Peruvian medical schools: Characteristics and addressed topics

**DOI:** 10.1016/j.heliyon.2023.e13320

**Published:** 2023-01-30

**Authors:** Christoper A. Alarcon-Ruiz, David R. Soriano-Moreno, Alvaro Taype-Rondan

**Affiliations:** a Neurociencia, Efectividad Clínica y Salud Pública, Universidad Científica del Sur, Lima, Peru; b EviSalud – Evidencias en Salud, Lima, Peru; c Unidad de Investigación Clínica y Epidemiológica, Universidad Peruana Unión, Lima, Peru; d Universidad San Ignacio de Loyola, Unidad de Investigación para la Generación y Síntesis de Evidencias en Salud, Lima, Peru

**Keywords:** Evidence-based medicine, Education, Medical, Undergraduate, Curriculum, Competency-based education, Peru

## Abstract

**Background:**

Medical schools are increasingly including evidence-based medicine (EBM) courses in their curricula. However, little is known about the characteristics of these courses in Peru. Therefore, the present study aimed to describe the characteristics and topics addressed by undergraduate courses on EBM in Peruvian medical schools, and to compare the content of these courses with predefined EBM competencies.

**Methods:**

We conducted a cross-sectional study. We obtained the syllabi of undergraduate EBM courses from all medical schools for the latest year available. We extracted their characteristics and categorized the topics they included according to the five steps necessary to apply EBM, divided into 22 competencies.

**Results:**

In 2021, Peru had 47 universities with active undergraduate medical schools, of which 9 (19.1%) had EBM courses. These courses were not mandatory in three of the universities, and were typically offered between the 2nd and 5th year of the degree program. When analysing the topics covered in the syllabi, we found that they addressed 7 to 13 of the 22 core competencies evaluated. The least addressed topics belonged to steps 4 (apply) and 5 (evaluate) of the EBM process.

**Conclusion:**

We found that few Peruvian universities offer EBM courses, and that these courses have heterogeneous characteristics, with syllabi that do not include all essential topics for applying the five steps of EBM.

## 1 Introduction

"Evidence-Based Medicine (EBM) is a rational approach to medical practice that involves critically reviewing available scientific evidence and applying it to the specific needs and circumstances of the patient [1]. To do this, EBM involves five sequential steps: formulating the question, obtaining the evidence, critically reviewing the evidence, making the decision, and evaluating the evidence [2]. In 2018, a multinational effort established 27 core competencies that health professionals should possess in order to practice EBM effectively [3].

In Peru, the Peruvian Association of Medical Schools describes that the clinical and public health competencies obtained by medical students during their studies must be based on the best available scientific evidence [4]. Similarly, The Ministry of Health indicates that the physician has to elaborate a rational treatment plan based on the best available evidence, and has to inform the patient about the efficacy and safety of the treatment [5]. However, a study conducted in 2017 on recently graduated physicians in Lima, Peru, reported that only one-third perceived having very good or excellent competencies to perform searches, critically read studies, or apply evidence to practice [6]. Other studies conducted among general practitioners, residents, and specialists in Peru also found a low perception of having competencies in different aspects of EBM [[7], [8], [9]].

In response to this gap, some medical schools in Peru have recently introduced EBM courses into their curricula [10]. These courses could help to establish an evidence-based medical practice in Peru, but for this to be achieved, their objectives and contents must be comprehensively aligned with the basic competencies in EBM expected of health professionals [3]. However, little is currently known about the characteristics and topics addressed by these EBM courses in Peru [11].

Therefore, the present study aims to describe the characteristics and topics covered by undergraduate EBM courses in Peruvian medical schools, and to compare the content of these courses with predefined EBM competencies.

## 2 Methods

### 2.1 Design and population

We conducted a cross-sectional study, evaluating the syllabi of undergraduate EBM courses in human medical schools in Peru. We included the syllabi for the years 2019, 2020, or 2021, according to the last year available.

We defined "EBM course" as any required or elective course in the undergraduate curriculum that explicitly stated in its name that it was an EBM course or whose main objective involves searching for and critically reading scientific studies in order to make health care decisions.

### 2.2 Procedures

In Peru, medical school programs typically last 7 years. The first two or three years cover basic sciences, while the following years focus on clinical sciences. The final year is a medical internship period. First, we identified the medical schools of those universities that were on the Superintendence of University Higher Education (SUNEDU, for its acronym in Spanish) Human Medicine Program Licensing schedule [12] and those identified in 2016 according to a previous report [13]. We then confirmed that each university had an active medical program by reviewing its website, and we obtained the most updated curricular grids available (between 2019 and 2021) through the same web platform. In those grids, we searched for courses named "Evidence-Based Medicine", "Critical Reading" or related. Then, we requested the syllabi of the pre-selected courses from student members of the scientific societies of medical students of each university. After reviewing the objectives, competencies, and content of the syllabi, we excluded courses that focused solely on epidemiology, research methodology, or critical reading of non-scientific texts.

### 2.3 Variables

For each medical school with an identified EBM course, we collected data on the type of institution (public or private), year of the start of the medical school, and location in Peru. Then, from the syllabi of each EBM course, we extracted information on the name of the course, year of the course in which it was taught, number of credits, type (theoretical, practical, or theoretical-practical), number of academic hours, number of teachers, syllabus, and final product of the course (final work or report used for evaluation).

### 2.4 Evaluation of competencies

For each course, we evaluated whether the syllabus or the final product of the course addressed any of the 22 core competencies corresponding to the five steps for applying EBM (ask, acquire, appraise and interpret, apply, and evaluate), established by a previously published consensus [3]. Compliance with each specific competency was considered if the syllabus mentioned at least one content of each specific competency in its syllabus or the final product of the course.

### 2.5 Statistical analysis

The data were analyzed descriptively, using frequencies and percentages, as well as tables of results.

## 3 Results

In 2021, Peru had 47 universities (26 private and 21 public) offering undergraduate medical programs, of which 9 (19.1%) had some course dedicated to EBM in their undergraduate curricula. According to their location: 3 of the 15 universities were located in Metropolitan Lima (Universidad Científica del Sur, Universidad Peruana Unión and Universidad Norbert Wiener), 1 of the 4 in Chiclayo (Universidad Señor de Sipán), 1 of the 2 in Cusco (Universidad Andina del Cusco), the only one in Ayacucho (Universidad Nacional San Cristobal de Huamanga), the only one in Pasco (Universidad Nacional Daniel Alcides Carrión), and one university that has campuses in Trujillo and Piura (Universidad Privada Antenor Orrego).

In three of the universities offering EBM courses, these courses were not mandatory. The courses were carried out in different years of the career (from 2nd to 5th year). Most of the courses were worth 2 or 3 academic credits, except the course at Señor de Sipan University, which was worth 5 credits and included 4 theoretical academic hours and 2 practical academic hours per week. In addition, five courses had only one teacher in charge, and one course had eight teachers in charge (Table 1).

**Table 1:** Characteristics of EBM courses taught in Peruvian medical schools.

University name	UAC	UCSUR	UNDAC	UNPRG	UNSCH	UNW	UPAO	UPEU	USS
City where the university's medical school is located.	Cusco	Lima	Pasco	Chiclayo	Ayacucho	Lima	Trujillo, Piura	Lima	Chiclayo
Type of institution	Private	Private	Public	Public	Public	Private	Private	Private	Private
Year of creation of medical school	2009	1998	2012	1981	2012	2016	1996 in Trujillo, 2011 in Piura	2012	2014
Course name	Evidence-based medicine	Evidence-based medicine	Evidence-based medicine. Bibliographic search strategies and critical analysis of medical literature.	Evidence-based medicine	Evidence-based medicine	Evidence-based medicine	Evidence-based medicine	Clinical research and critical reading	Evidence-based medicine
Elective or mandatory	Elective	Mandatory	Elective	Elective	Mandatory	Mandatory	Mandatory	Mandatory	Mandatory
Year of the course in which it is taught	4	5	5	3	4	5	2	4	3
Credits	3	2	3	2	3	2	2	2	5
Theoretical and practical hours per week	2, 2	1, 2	2, 2	1, 2	3, 0	1, 2	3^a^	3, 1	4, 2
Number of teachers	1	3	1	3	1	1	8	1	2
Final product of the course as part of the evaluation	Final report: Evaluates the methodology, interprets results, looks for biases, elaborates, and proposes a therapeutic alternative for a clinical case.	Final work: Protocol of scientific research and critical appraisal of scientific reading.	–	Final creditable product: PICO question, search strategy, bias assessment, significance assessment of the result, and applicability assessment.	–	A report in scientific journal format: Summary of a clinical case, differential diagnoses, and treatment.	–	–	Creditable product: Search strategy and critical reading analysis of a scientific article.
Number of competencies addressed	11/22	7/22	10/22	13/22	7/22	9/22	11/22	9/22	9/22

SUNEDU: Superintendence of University Higher Education; UAC: Universidad Andina del Cusco; UCSUR: Universidad Científica del Sur; UNDAC: Universidad Nacional Daniel Alcides Carrión; UNPRG: Universidad Nacional Pedro Ruiz Gallo; UNSCH: Universidad Nacional San Cristóbal de Huamanga; UNW: Universidad Norbert Wiener; UPAO: Universidad Privada Antenor Orrego; UPEU: Universidad Peruana Unión; USS: Universidad Señor de Sipán.

a It does not detail how many hours are theoretical and how many are practical.

When reviewing the syllabi of these EBM courses, we found that the courses addressed 7 to 13 of the 22 core competencies evaluated. The competencies most frequently addressed belonged to the first three steps of the EBM process (ask, acquire, and appraise/interpret), while the competencies related to the last two steps (apply and evaluate) were less frequently addressed.

Five core competencies were not addressed by any course (explaining the differences between background and foreground questions, obtaining the full text of articles, understanding the purpose and process of qualitative studies, practicing shared decision making, and overcoming knowledge translation barriers), and two were addressed by only one course (estimating individual expected benefit and audit for EBM) (Table 2 and Additional file 1).

**Table 2:** EBM core competencies addressed in the courses^a^.

Domains	Competencies	Courses that address it as a topic
1) Ask	1.1 Explain the difference between the types of questions that cannot typically be answered by research (background questions) and those that can (foreground questions)	0
	1.2 Identify different types of clinical questions, such as questions about treatment, diagnosis, prognosis, and etiology	6
	1.3 Convert clinical questions into structured, answerable clinical questions using PICO	6
2) Acquire	2.1 Outline the different major categories of sources of research information, including biomedical research databases or databases of filtered or pre-appraised evidence or resources	3
	2.2 Construct and carry out an appropriate search strategy for clinical questions	7
	2.3 State the differences in broad topics covered by the major research databases	4
	2.4 Outline strategies to obtain the full text of articles and other evidence resources	0
3) Appraise and interpret	3.1 Identify key competencies relevant to the critical evaluation of the integrity, reliability, and applicability of health-related research	3
	3.2 Interpret different types of measures of association and effect, including key graphical presentations	4
	3.3 Critically appraise and interpret a systematic review	7
	3.4 Critically appraise and interpret a treatment study	7
	3.5 Critically appraise and interpret a diagnostic accuracy study	8
	3.6 Distinguish evidence-based from opinion-based clinical practice guidelines	5
	3.7 Identify the key features of, and be able to interpret, a prognostic study	7
	3.8 Explain the use of harm and etiologies study for (rare) adverse effects of interventions	8
	3.9 Explain the purpose and processes of a qualitative study	0
4) Apply	4.1 Engage patients in the decision-making process, using shared decision-making, including explaining the evidence and integrating their preferences	0
	4.2 Outline different strategies to manage uncertainty in clinical decision-making in practice	3
	4.3 Explain the importance of baseline risk of individual patients when estimating the individual expected benefit	1
	4.4 Interpret the grading of the certainty in evidence and the strength of recommendations in health care	6
5) Evaluate	5.1 Recognize potential individual-level barriers to knowledge translation and strategies to overcome these	0
	5.2 Recognize the role of personal clinical audit in facilitating evidence-based practice	1

a Competencies were taken from Albarqouni L et al. Core Competencies in Evidence-Based Practice for Health Professionals: Consensus Statement Based on a Systematic Review and Delphi Survey. JAMA Netw Open. 2018; 1 (2):e180281 [3].

## 4 Discussion

We found that 9 universities with medical schools in Peru had EBM courses. These courses had heterogeneous characteristics, and their syllabi do not make explicit that all the relevant competencies of each step of EBM were addressed, especially concerning the last two steps (apply and evaluate).

To get general practitioners to apply EBM in their practice, it is necessary that they acquire these competencies during undergraduate. However, we found that in 2021 only 9 of the 47 medical schools in Peru had some EBM course. This is similar to what was found in Italy in 2017 where 8 of the 40 medical schools had some EBM course [14]. On the other hand, it is lower than what was found in medical schools in Canada and the United States in 2011 where 95% of the schools had some EBM course in their curriculum [15]. In Peru, the low number of medical schools with EBM courses is possibly due to the fact that they are local initiatives, but not national, as was observed in a study conducted in Mexico in 2021 [16].

It should be kept in mind that when applying an EBM course, it is essential that students acquire both theoretical and practical competencies of the 5 steps of EBM [3]. Therefore, it is important that EBM teaching be carried out in an orderly and structured manner. However, it is still common to find that EBM teaching is not structured or does not pursue defined competencies [14,15].

In evaluating EBM course syllabi, we found that the medical schools evaluated focused on the first three steps of EBM: 1) question formulation, 2) search, and 3) interpretation of scientific studies, similar to medical schools in the United Kingdom in 2007 [17]. Concordantly, a systematic review that evaluated different EBM teaching interventions described that most of these focused on critical reading of studies (step 3), and very few included all five steps [18]. This prioritization of certain content may be due to a misconception that EBM only focuses on knowing how to find and read scientific articles, without considering the importance of decision-making and its revaluation [19,20]. This in turn could affect the concept that students, professionals, and teachers have of EBM and decision making.

The apply (step 4) and evaluate (step 5) steps of EBM require competencies that have been virtually unaddressed by courses in Peru, which differs from a recent scoping review that found that most EBM curricula for physicians in training address all 5 steps of EBM [21]. Steps 4 and 5 include how to perform shared decision-making processes with the patient, manage uncertainty in clinical decision-making in practice, assess the patient individual risk when estimating individual benefits, overcome individual-level barriers to knowledge translation, and personal clinical audit. These competencies are not intuitive, as they require the student to understand concepts and handle certain tools (such as decision aids, or Evidence-to-Decision frameworks) [6]. Underestimating these competencies would be a mistake, since they are indispensable for making evidence-based decisions, and some experts suggest that it may be more useful to teach students to use pre-processed evidence than to teach them to read primary studies [22].

In Peru, although health professionals seem to have a favorable disposition to learn and apply EBM, they recognize that lack of time, inadequate training, and lack of access to or understanding of the studies are important limitations [7,8,23]. In addition, clinical practice in hospitals may not be evidence-based [7]. These contextual limitations should be addressed by medical schools to ensure that graduates apply EBM to their practice.

In general, EBM teaching is limited by the low number of hours and credits which are minimally representative of the total curriculum. In addition, there is a shortage of trained tutors and teaching materials for learning EBM. More resources are needed so that teachers can be trained in this topic, since these are scarce, especially in Spanish. It is necessary to standardize at the national or local level what EBM competencies students should acquire, as well as the minimum curricular content required for this purpose. There are Latin American experiences in this regard, such as the standardization of EBM curricular content in Brazil [24].

Ideally, EBM integration should be longitudinal throughout all years of the degree program [25]. This could be achieved using spiral curricular designs, so that basic EBM concepts are introduced in the early years, reviewed frequently during subsequent courses, and become increasingly complex as students progress through the career [25,26]. Concordantly, other authors have proposed that educational interventions that started early in the career but progressively developed over the following years, can improve competencies, knowledge, and attitudes toward EBM in students [[27], [28], [29]].

It should be taken into account that the final objective of EBM teaching is that the student can apply it in real clinical scenarios, so it is important to integrate EBM into other curricular activities (e.g., hospital rotations). In addition, it has been suggested that EBM course evaluations should be based on simulation exercises or the objective structured clinical examination (OSCE) [15]. To increase student interest in EBM, its topics could be included in national evaluations [30] such as the National Medical Student Examination (ENAM) and the National Medical Residency Admission Examination (ENARM). Finally, it is important to assess the knowledge and competencies in EBM with which physicians graduate, using standardized tools [31].

### 4.1 Limitations and strengths

We obtained the syllabi through the websites and by requesting them from students, which may have led to outdated syllabi being included. We only evaluated those syllabi with explicit EBM content, without considering EBM content that may have been incorporated into other research-related or clinical courses. However, research courses in Peruvian medical schools generally do not involve EBM concepts beyond those related to biostatistics or epidemiology [32]. In addition, although the hidden curriculum within formal education was not evaluated, it is likely to involve heterogeneous competencies that do not usually include EBM concepts for decision making [33].

We considered that a course had addressed a competency when its syllabus included a topic that appeared to be related to that competency. Therefore, it is possible that the course only addressed a part of the topic, and the competency was not fully formed. However, it is also possible that a course does address a competency, but it does not appear in its syllabus in an identifiable way. Future studies should evaluate how these courses are being implemented and how successful they are in imparting the desired competencies.

Despite these limitations, this study is one of the first to analyse EBM teaching in medical education in Peru.

## 5 Conclusion

In conclusion, few Peruvian medical schools had dedicated EBM courses. Furthermore, the syllabi of these courses did not cover all the necessary topics to ensure that students acquire the essential competencies for decision making.

## Declarations

### Author contribution statement

Christoper A. Alarcon-Ruiz; Alvaro Taype-Rondan: Conceived and designed the experiments; Performed the experiments; Analyzed and interpreted the data; Contributed reagents, materials, analysis tools or data; Wrote the paper.

David R. Soriano-Moreno: Analyzed and interpreted the data; Contributed reagents, materials, analysis tools or data; Wrote the paper.

### Funding statement

This research did not receive any specific grant from funding agencies in the public, commercial, or not-for-profit sectors.

### Data availability statement

Data included in article/supp. material/referenced in article.

### Declaration of interest’s statement

The authors declare the following conflict of interests: ATR has worked for the Universidad Científica del Sur. CAAR is currently a teaching assistant in the Evidence-based medicine course of the Universidad Científica del Sur.

DRSM is a medical student of the Universidad Peruana Unión.

## References

[1] Sackett D.L., Rosenberg W.M.C., Gray J.A.M., Haynes R.B., Richardson W.S. (1996). Evidence based medicine: what it is and what it isn't. BMJ.

[2] Guyatt G.H., Rennie D., Meade M.O., Cook D.J. (2015).

[3] Albarqouni L., Hoffmann T., Straus S., Olsen N.R., Young T., Ilic D., Shaneyfelt T., Haynes R.B., Guyatt G., Glasziou P. (2018). Core competencies in evidence-based practice for health professionals: consensus statement based on a systematic review and Delphi survey. JAMA Netw. Open.

[4] Salcedo Espinoza C.G. (2019). Asociación Peruana de Facultades de Medicina. Competencias esenciales a lograr en el internado de medicina.

[5] de Salud del Perú Ministerio Documento técnico: perfil de competencias esenciales que orientan la formación de los profesionales médicos de la salud, (n.d.). https://www.gob.pe/institucion/minsa/normas-legales/1364189-960-2020-minsa.

[6] Nieto-Gutierrez W., Zafra-Tanaka J.H., Pacheco-Barrios K., Taype-Rondan A. (2020). Self-perception of competences in clinical practice among recently graduated physicians from Lima, Peru. Heliyon.

[7] Segundo-Paredes J.A., Gonzales-Medina C.A., Francia-De-la-Cruz R.J., Valdivia-Vera E., Mejia-Veramendi J.P., Arango-Ochante P.M. (2019). Conocimientos, actitudes y prácticas de la medicina basada en evidencias en médicos asistentes de un hospital público. Lima- Perú, Rev Peru Investig Matern Perinat..

[8] Canelo Aybar C.G., Santos Alarcon, Amao Ruiz E.J., Beteta Vejarano V.S., Monge Salgado E. (2007). Conocimientos, actitudes y prácticas de la medicina basada en evidencias en médicos asistentes y residentes en dos hospitales de Lima-Perú. Rev. Méd. Hered..

[9] Romero-Robles M.A., Soriano-Moreno D.R., García-Gutiérrez F.M., Condori-Meza I.B., Sing-Sánchez C.C., Bulnes Alvarez S.P., Alarcon-Ruiz C.A., Taype-Rondan A., Viteri‐García A. (2022). Self-perceived competencies on evidence-based medicine in medical students and physicians registered in a virtual course: a cross-sectional study. Med. Educ. Online.

[10] Fernández-Guzman D., Campero-Espinoza A.B., Ccorahua-Rios M.S., Medina-Quispe C.I., Chávez-Cruzado E., Galvez-Olortegui J. (2021). De la evidencia a la decisión: La necesidad de competencias en Medicina Basada en Evidencias en escuelas de medicina peruanas, para la toma de decisiones clínicas. Rev. Cuerpo Méd. Hosp. Nac. Almanzor Aguinaga Asenjo..

[11] Domecq J.P., Prutzky G., Málaga G. (2013). La implementación y el uso integral de la medicina basada en la evidencia: aún pendientes e indispensables. Rev. Peru. Med. Exp. Salud Pública.

[12] Superintendencia de Educación Superior Universitaria (2021). https://www.sunedu.gob.pe/licenciamiento-programas-medicina-humana-cronograma/.

[13] Mayta-Tristán P., Cuentas M., Núñez-Vergara M. (2016). Responsabilidad de las instituciones ante la proliferación de escuelas de medicina en el Perú. Acta Méd. Peru..

[14] Antonino C., Andrea C., Mosti M., Giuseppe A., Elena C., Matteo C., Sonia C., Giulia D.M.T., Nicola M., Adolfo M. (2018). 44 Teaching of evidence-based medicine in Italian medical schools: a systematic analysis of courses and syllabi. BMJ Evid.-Based Med..

[15] Blanco M.A., Capello C.F., Dorsch J.L., (Jerry) Perry G., Zanetti M.L. (2014). A survey study of evidence-based medicine training in US and Canadian medical schools. J. Med. Libr. Assoc. JMLA..

[16] Rodriguez D., Martinez-Alvarado J.D., Garcia-Toto R., Genel-Rey T.I. (2022). Teaching evidence-based medicine in Mexico: a systematic review of medical doctor curriculums at a national level. BMJ Evid.-Based Med..

[17] Meats E., Heneghan C., Crilly M., Glasziou P. (2009). Evidence-based medicine teaching in UK medical schools. Med. Teach..

[18] Albarqouni L., Hoffmann T., Glasziou P. (2018). Evidence-based practice educational intervention studies: a systematic review of what is taught and how it is measured. BMC Med. Educ..

[19] Siwek J. (2018). Evidence-based medicine: common misconceptions, barriers, and practical solutions. Am. Fam. Physician.

[20] Poolman R.W., Petrisor B.A., Marti R.K., Kerkhoffs G.M., Zlowodzki M., Bhandari M. (2007). Misconceptions about practicing evidence-based orthopedic surgery. Acta Orthop..

[21] Halalau A., Holmes B., Rogers-Snyr A., Donisan T., Nielsen E., Cerqueira T.L., Guyatt G. (2021). Evidence-based medicine curricula and barriers for physicians in training: a scoping review. Int. J. Med. Educ..

[22] Lehane E., Leahy-Warren P., O'Riordan C., Savage E., Drennan J., O'Tuathaigh C., O'Connor M., Corrigan M., Burke F., Hayes M., Lynch H., Sahm L., Heffernan E., O'Keeffe E., Blake C., Horgan F., Hegarty J. (2019). Evidence-based practice education for healthcare professions: an expert view. BMJ Evid.-Based Med..

[23] Tomatis C., Taramona C., Rizo-Patrón E., Hernández F., Rodríguez P., Piscoya A., Gonzales E., Gotuzzo E., Heudebert G., Centor R.M., Estrada C.A. (2011). Evidence-based medicine training in a resource-poor country, the importance of leveraging personal and institutional relationships. J. Eval. Clin. Pract..

[24] Araujo G.A., Correia L.C.L., Siqueira J.R., Nogueira L.C., Meziat-Filho N., Costa L.O.P., Reis F.J. (2021). Consensus on evidence-based medicine curriculum contents for healthcare schools in Brazil. BMJ Evid.-Based Med..

[25] Maggio L.A., ten Cate O., Chen H.C., Irby D.M., O'Brien B.C. (2016). Challenges to learning evidence-based medicine and educational approaches to meet these challenges: a qualitative study of selected EBM curricula in U.S. And Canadian medical schools. Acad. Med..

[26] Elçin M., Turan S., Odabaşı O., Sayek İ. (2014). Development and evaluation of the evidence-based medicine program in surgery: a spiral approach. Med. Educ. Online.

[27] Kotur P.F. (2012). Introduction of evidence-based medicine in undergraduate medical curriculum for development of professional competencies in medical students. Curr. Opin. Anesthesiol..

[28] Young T., Rohwer A., Volmink J., Clarke M. (2014). What are the effects of teaching evidence-based health care (EBHC)? Overview of systematic reviews. PLoS One.

[29] West C.P., Jaeger T.M., McDonald F.S. (2011). Extended evaluation of a longitudinal medical school evidence-based medicine curriculum. J. Gen. Intern. Med..

[30] Rashid A., Finnikin S., Tackett S. (2021). Accreditation drives teaching: evidence-based medicine and medical education standards. BMJ Evid.-Based Med..

[31] Kumaravel B., Hearn J.H., Jahangiri L., Pollard R., Stocker C.J., Nunan D. (2020). A systematic review and taxonomy of tools for evaluating evidence-based medicine teaching in medical education. Syst. Rev..

[32] Taype-Rondan A., Huaccho-Rojas J., Pereyra-Elias R., Mayta-Tristán P., Mejia C.R. (2015). Características de los cursos de investigación en escuelas de medicina del Perú. Arch. Med..

[33] Braschi E., Stacey D., Légaré F., Grad R., Archibald D. (2020). Evidence-based medicine, shared decision making and the hidden curriculum: a qualitative content analysis. Perspect. Med. Educ..

