# Supplementary material for: Undergraduate courses of evidence-based medicine in Peruvian medical schools: Characteristics and addressed topics
Source: Heliyon. 2023 Jan 30;9(2):e13320. doi: 10.1016/j.heliyon.2023.e13320 (PMC9932740; doi:10.1016/j.heliyon.2023.e13320)
Supplement: Multimedia component 1 [file mmc1.docx]

## Additional file 1. EBM competencies addressed in the evaluated courses, detailing the topics mentioned in each syllabus.

| **EBM domains** | **Core competencies** | **UAC** | **UCSUR** | **UNDAC** | **UNPRG** | **UNSCH** | **UNW** | **UPAO** | **UPeU** | **USS** |
| --- | --- | --- | --- | --- | --- | --- | --- | --- | --- | --- |
| 1) Ask | 1.1 Explain the difference between the types of questions that cannot typically be answered by research (background questions) and those that can (foreground questions) |  |  |  |  |  |  |  |  |  |
|  | 1.2 Identify different types of clinical questions, such as questions about treatment, diagnosis, prognosis, and etiology | Formulation of answerable clinical questions | Research question formulation |  |  |  | Specific answerable questions on diagnosis, prognosis, treatment, or intervention | Structure of the research question | Formulation of the research question | Structured, clinical, and answerable questions. |
|  | 1.3 Convert clinical questions into structured, answerable clinical questions using PICO | PICO mnemonic | Research question: PICO |  | PICO question to solve a clinical question |  | PICO System: Patients, Intervention / Comparison, and Outcomes | PICO/PECO strategies |  | PICO question formulation and question types |
| 2) Acquire | 2.1 Outline the different major categories of sources of research information, including biomedical research databases or databases of filtered or pre-appraised evidence or resources |  |  |  | Bibliographic bases |  | Evidence-based medicine data sources |  |  | Information sources, electronic databases, and reference web librarianship |
|  | 2.2 Construct and carry out an appropriate search strategy for clinical questions | Integration of the PICO mnemonic to the search for answers. Advanced search for biomedical information | Bibliographic search workshop |  | Bibliographic search: Boolean operators and search strategy. | Computerized bibliographic search | Obtaining the original studies |  | Information search I and II | Search strategies |
|  | 2.3 State the differences in broad topics covered by the major research databases | Basic search for information: use of Pubmed, Cochrane, ScienceDirect, Scielo, Google Scholar. | Pubmed and Google Scholar | Information on high-impact factor in Scopus and Web of Science |  |  |  | Presentation of Scielo, Latindex, Pubmed, Scopus and WoS, |  |  |
|  | 2.4 Outline strategies to obtain the full text of articles and other evidence resources |  |  |  |  |  |  |  |  |  |
| 3) Appraise and interpret | 3.1 Identify key competencies relevant to the critical evaluation of the integrity, reliability, and applicability of health-related research | Biases in biomedical research |  | Internal and external validity. Reliability, random and systematic error, and biases in the research process. | Biases in a research article. Hypothesis testing, p-value, and statistical inference. |  |  |  |  |  |
|  | 3.2 Interpret different types of measures of association and effect, including key graphical presentations | Measures of association and impact, hypothesis tests, tests for comparing means or proportions |  | Absolute risk, relative risk, and attributable risk factor. Risk reduction. | Risk indicators: Odds Ratio, Relative Risk. Measures of magnitude of effect: Number needed to treat, absolute risk reduction, relative risk reduction. |  |  | Interpretation of measures of association (Prevalence Ratio, Odds Ratio, and Relative Risk). |  |  |
|  | 3.3 Critically appraise and interpret a systematic review | Critical reading of SR articles |  | Narrative reviews, SRs, and meta-analyses | Critical reading of SR and meta-analysis | Critical Reading of Scientific Articles: Meta-Analysis |  | Definition of SR and meta-analysis, the difference with a narrative review. Basic notions of heterogeneity and meta-analysis. | Meta-analysis | Literature review and SR, meta-analysis, and how to apply SR results. |
|  | 3.4 Critically appraise and interpret a treatment study | Critical reading of treatment articles |  |  | Critical reading of an intervention study | Critical Reading of Scientific Articles: Clinical Trials | RCTs and phases, magnitude and estimation of the effect of treatments. | Design and phases of an RCT according to the FDA. Mention of basic aspects of quality assessment with Cochrane RoB 2. Presentation of the CONSORT guide | Critical reading on a treatment | Study designs, data analysis, and guidelines for the development of a critical reading analysis of a prevention and treatment article. |
|  | 3.5 Critically appraise and interpret a diagnostic accuracy study | Critical reading of diagnostic articles. Validity of diagnostic tests |  | Reading of diagnostic studies: Reference standard, positive predictive value, negative predictive value, sensitivity, specificity, likelihood ratio, ROC curve. | Statistical analysis in diagnostic tests: Sensitivity, specificity, positive predictive value, negative predictive value, likelihood ratio, ROC curve. Critical reading of diagnostic studies | Critical reading of scientific articles: Validity of diagnostic tests. | To evaluate the capacity, validity, and applicability of diagnostic tests in clinical practice, considering their cost-utility. | Sensitivity, specificity, positive predictive value, and negative predictive value. Presentation of the STARD guide | Critical reading of a study on differential diagnosis. | Design of diagnostic test studies, sensitivity, specificity, and likelihood ratios. |
|  | 3.6 Distinguish evidence-based from opinion-based clinical practice guidelines |  | Critical reading with AGREE-2 | Types of CPG, development, and steps for its elaboration. Critical reading with AGREE-2 | Critical reading of a CPG |  |  | Basic notions of CPGs: Definitions, uses, importance, applications | Critical reading of a CPG |  |
|  | 3.7 Identify the key features of, and be able to interpret, a prognostic study |  |  | Most suitable tools for determining prognosis, Hazard Ratio, Kaplan Meier Curve | Critical reading of prognostic studies | Critical Reading of Scientific Articles: Prognostic Factors | EBM to assess prognosis | Definition and uses of prognostic studies. Presentation of a Kaplan-Meyer curve. | Critical reading on the prognosis | Guidelines for the development of critical reading analysis of a prognostic studies article. |
|  | 3.8 Explain the use of harm and etiologies study for (rare) adverse effects of interventions | Critical reading of epidemiological articles | Critical reading with STROBE | Tools best suited to determine etiology | Critical reading of harm studies | Critical Reading of Scientific Articles: Risk Factors |  | Observational studies: Cross-sectional, case-control, and cohort with STROBE. | Critical reading of a study on the harmful effects of an exposure. | Research designs and critical reading for risk identification, and estimation of injury rates and risks. |
|  | 3.9 Explain the purpose and processes of a qualitative study |  |  |  |  |  |  |  |  |  |
| 4) Apply | 4.1 Engage patients in the decision-making process, using shared decision-making, including explaining the evidence and integrating their preferences |  |  |  |  |  |  |  |  |  |
|  | 4.2 Outline different strategies to manage uncertainty in clinical decision-making in practice |  |  | Decision-making and diagnostic and therapeutic reasoning. |  | A clinical case study using a problem-oriented methodology | Cognitive autopsy of a clinical case |  |  |  |
|  | 4.3 Explain the importance of baseline risk of individual patients when estimating the individual expected benefit |  |  |  | Application of EBM to the solution of clinical questions: Applicability taking into account treatment availability, cost, risk, and patient values. |  |  |  |  |  |
|  | 4.4 Interpret the grading of the certainty in evidence and the strength of recommendations in health care | Grade of recommendation scales and level of evidence classification. GRADE system to make recommendations for clinical practice | Critical reading with GRADE | Levels of evidence quality. Level of recommendations. | GRADE Methodology |  |  | Basic notions and important aspects of the GRADE methodology | GRADE Methodology |  |
| 5) Evaluate | 5.1 Recognize potential individual-level barriers to knowledge translation and strategies to overcome these |  |  |  |  |  |  |  |  |  |
|  | 5.2 Recognize the role of personal clinical audit in facilitating evidence-based practice |  |  |  |  |  | EBM and self-assessment as a feedback strategy. |  |  |  |

AGREE: Appraisal of Guidelines for Research and Evaluation; CONSORT: Consolidated Standards of Reporting Trials; FDA: U.S. Food and Drug Administration; RCT: Randomized controlled trial, CPG: Clinical practice guideline; GRADE: The Grading of Recommendations Assessment, Development, and Evaluation; EBM: Evidence based-medicine; PICO: Population, intervention, comparator and outcome; RoB: Cochrane tool for risk of bias assessment; SR: Systematic review; STARD: Reporting Guidelines for Diagnostic Accuracy Studies; STROBE: Strengthening the Reporting of Observational Studies in Epidemiology; UAC: Universidad Andina del Cusco; UCSUR: Universidad Científica del Sur; UNDAC: Universidad Nacional Daniel Alcides Carrión; UNPRG: Universidad Nacional Pedro Ruiz Gallo; UNSCH: Universidad Nacional San Cristóbal de Huamanga; UNW: Universidad Norbert Wiener; UPAO: Universidad Privada Antenor Orrego; UPeU: Universidad Peruana Unión; USS: Universidad Señor de Sipán.
